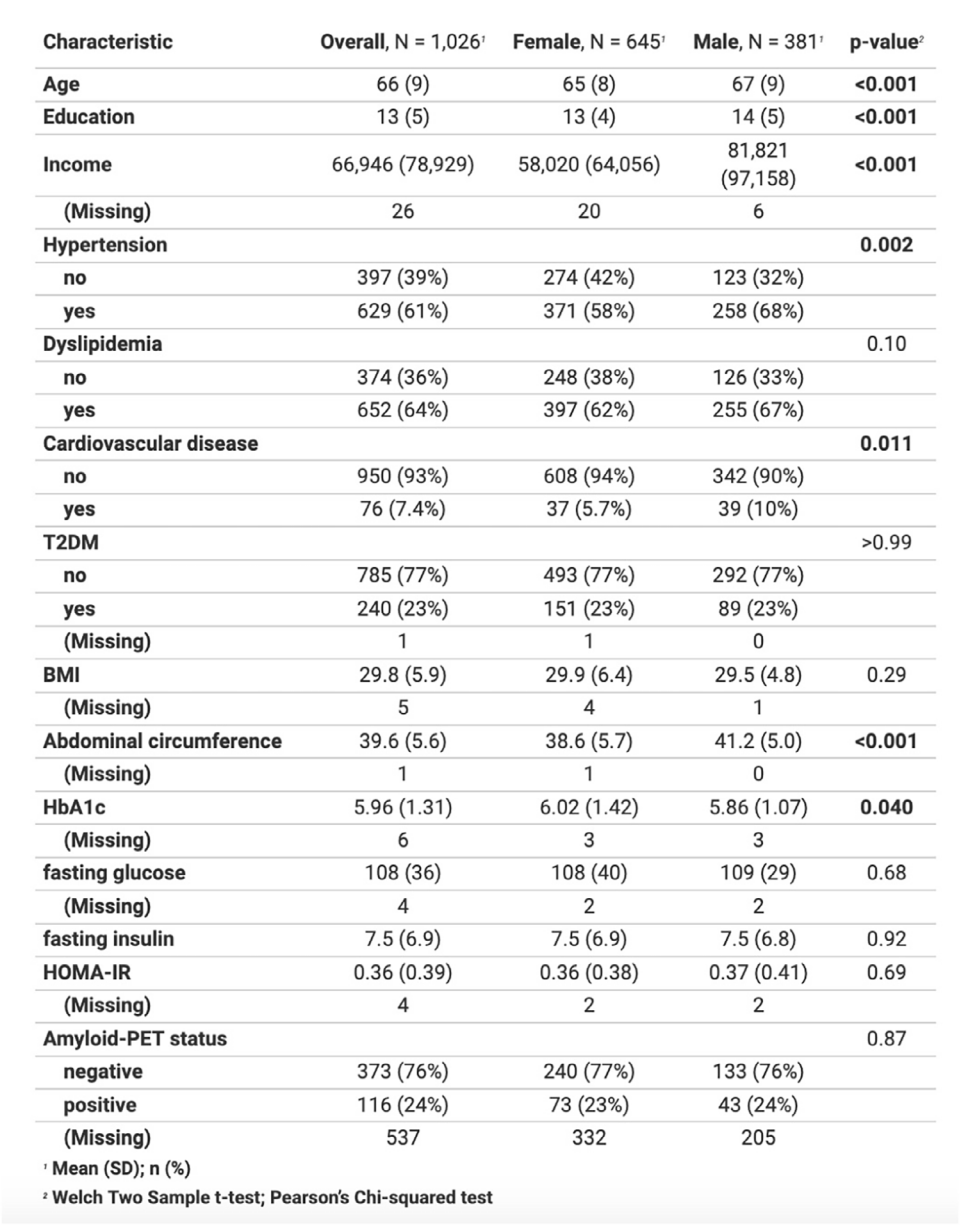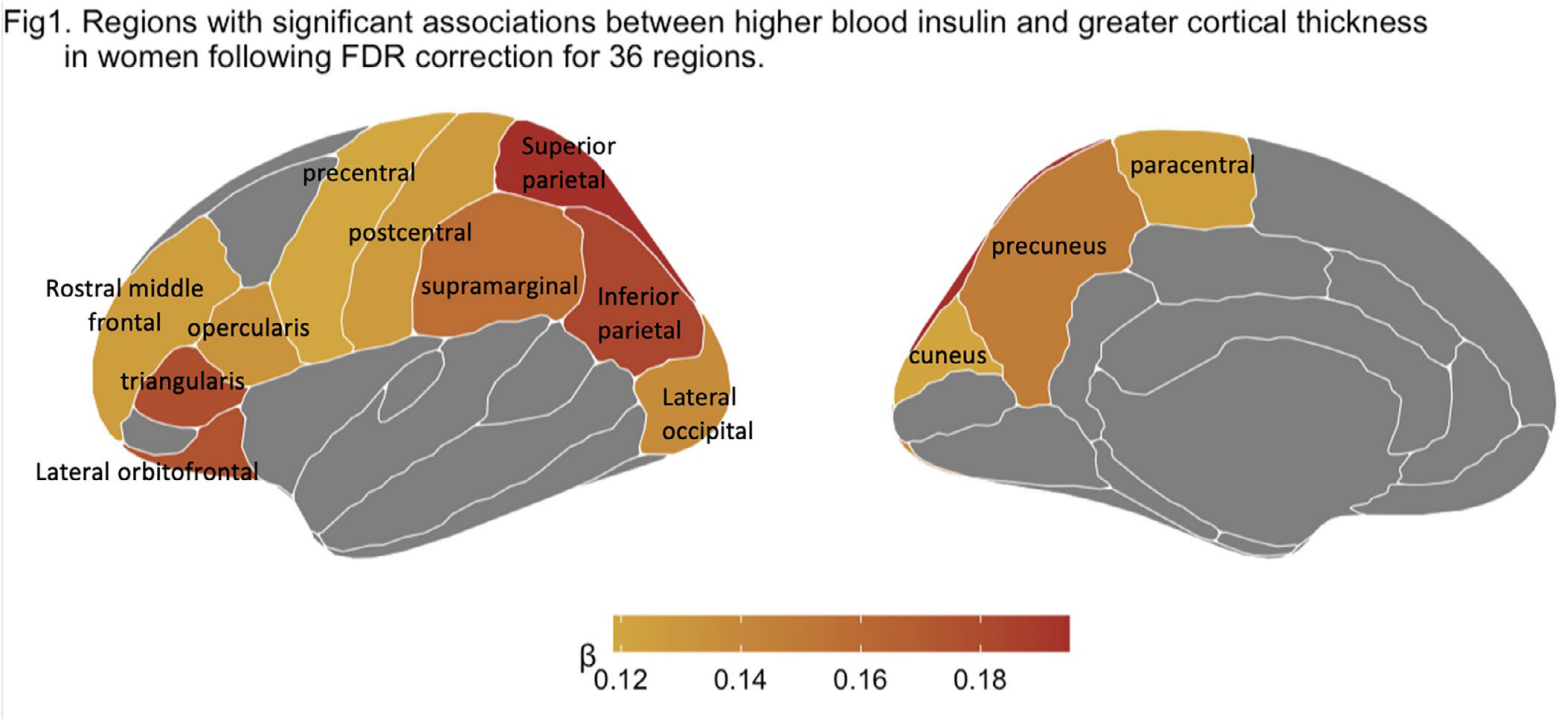# The role of sex in the association between peripheral insulin and cortical thickness in cognitively intact older adults by amyloid status

**DOI:** 10.1002/alz.091554

**Published:** 2025-01-09

**Authors:** Amaryllis A Tsiknia, Meral A Tubi, Noelle Lee, Brandon J Hall, Melissa Petersen, Stephanie E Large, Arthur W. Toga, Sid E. O'Bryant, Kristine Yaffe, Meredith N Braskie

**Affiliations:** ^1^ Mark and Mary Stevens Neuroimaging and Informatics Institute, Keck School of Medicine, University of Southern California, Los Angeles, CA USA; ^2^ Stevens Neuroimaging and Informatics Institute, Los Angeles, CA USA; ^3^ Translational Neuroimaging Laboratory, The McGill University Research Centre for Studies in Aging, Montréal, QC Canada; ^4^ University of North Texas Health Science Center, Fort Worth, TX USA; ^5^ Laboratory of Neuro Imaging, Stevens Neuroimaging and Informatics Institute, Keck School of Medicine, University of Southern California, Los Angeles, CA USA; ^6^ University of California, San Francisco, Weill Institute for Neurosciences, San Francisco, CA USA

## Abstract

**Background:**

Compared to men, insulin‐resistant women have more brain atrophy, cognitive impairment, and higher dementia risk, but the mechanisms for this sex difference are unknown. We examined sex differences in how blood insulin relates to cortical thickness in cognitively intact older adults. Gaining a better understanding of the relationship between insulin and brain health could help inform the development of insulin‐targeting treatments and reduce the risk of dementia associated with insulin resistance.

**Method:**

We performed MRI and florbetaben (amyloid PET) scanning and acquired fasting blood insulin on 1026 cognitively normal participants from the Health & Aging Brain Study – Health Disparities (50‐90 years; 645 female) (Table 1). Amyloid positivity (AB+) was defined as global SUVR>1.08, using a whole cerebellum reference region. We used linear regressions to relate blood insulin to cortical thickness in 34 mean bilateral Freesurfer‐derived brain regions and left and right hippocampal volume (FDR‐corrected for 36 regions). We additionally conducted separate linear regressions within each sex and tested for a sex*insulin interaction on cortical thickness and hippocampal volume. Covariates included age, sex, blood glucose levels, abdominal circumference, years of education, and income.

**Result:**

In the full sample, higher insulin was associated with thicker superior parietal (β=0.11, p‐corrected=0.02), inferior parietal (β=0.10, p‐corrected=0.03) and lateral occipital cortex (β=0.12, p‐corrected=8.14e‐03). In women, but not men, higher insulin was associated with thicker cortex in several frontal, parietal, and occipital regions (Figure 1). A sex*insulin interaction on cortical thickness was significant in the pars triangularis (β=‐0.18, p‐corrected=0.04), lateral orbitofrontal (β=‐0.23, p‐corrected=6.80e‐03), superior parietal (β=‐0.17, p‐corrected=0.04), and inferior parietal cortex (β=‐0.18, p‐corrected=0.02), even after controlling for hypertension and cardiovascular disease presence, which differed between sexes. Higher insulin was associated with thicker cortex in several frontal and parietal regions in AB‐ but not AB+ women, with an amyloid status*insulin interaction in the pars orbitalis (β=‐0.42, p‐corrected=0.03).

**Conclusion:**

Higher blood insulin correlates with thicker cortex in cognitively intact women, particularly those who are AB‐, but not in men. Future research on how sex hormones affect insulin signaling and the potential modulating role of amyloid could further our understanding of neurodegenerative processes and guide targeted interventions in dementia prevention.